# EABI-DETR: An Efficient Aerial Small Object Detection Network

**DOI:** 10.3390/biomimetics10110770

**Published:** 2025-11-13

**Authors:** Fufang Li, Yuehua Zhang, Yuxuan Fan

**Affiliations:** School of Computer Science and Cyber Engineering, Guangzhou University, Guangzhou 510006, China; yuehua@e.gzhu.edu.cn (Y.Z.); fanyx@e.gzhu.edu.cn (Y.F.)

**Keywords:** UAV object detection, multi-scale feature fusion, aerial images, RT-DETR, EMA

## Abstract

Small object detection, as an important research topic in computer vision, has been widely applied in aerial visual tasks such as remote sensing and UAV imagery. However, due to challenges such as small object size, large-scale variations, and complex backgrounds, existing detection models often struggle to capture fine-grained semantics and high-resolution texture information in aerial scenes, leading to limited performance. To address these issues, this paper proposes an efficient aerial small object detection model, EABI-DETR (Efficient Attention and Bi-level Integration DETR), based on the RT-DETR framework. The proposed model introduces systematic enhancements from three aspects: (1) A lightweight backbone network, C2f-EMA, is developed by integrating the C2f structure with an efficient multi-scale attention (EMA) mechanism. This design jointly models channel semantics and spatial details with minimal computational overhead, thereby strengthening the perception of small objects. (2) A P2-BiFPN bi-directional multi-scale fusion module is further designed to incorporate shallow high-resolution features. Through top-down and bottom-up feature interactions, this module enhances cross-scale information flow and effectively preserves the fine details and textures of small objects. (3) To improve localization robustness, a Focaler-MPDIoU loss function is introduced to better handle hard samples during regression optimization. Experiments conducted on the VisDrone2019 dataset demonstrate that EABI-DETR achieves 53.4% mAP@0.5 and 34.1% mAP@0.5:0.95, outperforming RT-DETR by 6.2% and 5.1%, respectively, while maintaining high inference efficiency. These results confirm the effectiveness of integrating lightweight attention mechanisms and shallow feature fusion for aerial small object detection, offering a new paradigm for efficient UAV-based visual perception.

## 1. Introduction

### 1.1. Motivations

With the rapid development of unmanned aerial vehicle (UAV) technology, aerial imagery has become an essential data source for intelligent visual tasks, finding broad applications in military reconnaissance [1], traffic monitoring [2], disaster rescue [3], pedestrian detection [4], and agricultural management [5]. However, unlike natural scenes, objects in aerial images are typically small in scale, densely distributed, and easily confused with complex backgrounds. These characteristics cause small targets to be easily overwhelmed by local textures or noise in the feature space, making it difficult to extract discriminative representations and posing significant challenges for aerial small object detection.

The emergence of deep learning has driven remarkable progress in object detection. Existing methods can be broadly categorized into two-stage detectors (e.g., the R-CNN series [6,7,8]) and one-stage detectors (e.g., SSD [9] and the YOLO series [10,11,12]). The former achieves high accuracy but suffers from complex architectures and slow inference, whereas the latter excels in real-time detection but is constrained by the limited receptive field of convolutional operations, making it difficult to simultaneously capture global contextual dependencies and fine-grained small-object details.

**Figure 1:**
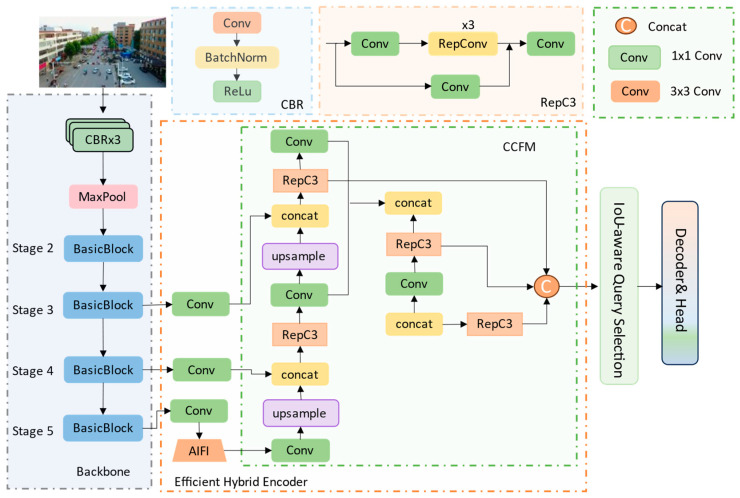
The overall architecture of the RT-DETR network. The model adopts an efficient hybrid encoder that combines Transformer-based intra-scale feature interaction (AIFI) with CNN-based cross-scale feature-fusion module (CCFM) to extract multi-scale features. Subsequently, an IoU-aware query selection module generates a fixed number of initial object queries, which are iteratively refined by the decoder to predict object classes and bounding boxes. RepConv denotes the re-parameterized convolution.

The introduction of Transformers has provided a new perspective for object detection. DETR-based models [13,14,15] reformulate object detection as a set prediction problem, leveraging the self-attention mechanism to model global dependencies while eliminating the need for non-maximum suppression (NMS) in post-processing. However, DETR-like detectors still suffer from slow convergence, high computational cost, and insufficient feature utilization. Building upon this foundation, RT-DETR [16] enhances real-time performance by introducing the intra-scale feature interaction (AIFI) and the cross-scale feature-fusion module (CCFM), as illustrated in Figure 1. Nevertheless, it performs detection only on the P3, P4, and P5 feature levels, neglecting shallow high-resolution features (P2). This omission limits its ability to capture fine-grained details and micro-scale object textures, thereby leading to a noticeable performance bottleneck.

Notably, physiological studies [17] have shown that the human visual system extensively leverages contextual information to facilitate object search in natural scenes. Through multi-level perception and top-down selective attention mechanisms, humans can rapidly focus on salient targets while suppressing redundant background interference. This biologically inspired mechanism of hierarchical feature integration and attentional modulation provides valuable insights for enhancing multi-scale perception and feature focusing capabilities in visual detection models.

Therefore, a critical challenge in aerial small object detection is how to maintain high inference efficiency while effectively combining the local representational capacity of CNN with the global modeling capability of Transformer. Moreover, it remains essential to exploit attention mechanisms for selective feature enhancement and to fully utilize shallow high-resolution features to strengthen fine-grained representations and multi-scale saliency perception an issue that remains central to advancing aerial small object detection.

### 1.2. Contributions

To address the limitations of RT-DETR in utilizing shallow features and perceiving small objects, this paper proposes an efficient aerial small object detection model named EABI-DETR. Inspired by the hierarchical attention mechanism of biological vision, the proposed approach systematically improves RT-DETR from three perspectives multi-scale representation enhancement, shallow-feature fusion, and localization robustness optimization. The major contributions are summarized as follows:

1. C2f-EMA lightweight feature extraction module: The Efficient Multi-scale Attention (EMA) mechanism is embedded into the C2f structure to achieve adaptive fusion of channel semantics and spatial details, resulting in an efficient and lightweight multi-scale feature representation. This design significantly enhances the model’s ability to perceive the position and boundary of small objects.
2. P2-BiFPN multi-scale fusion structure: On the basis of the Bi-directional Feature Pyramid Network (BiFPN) framework, a shallow high-resolution feature path (P2) is explicitly introduced to replace the original CCFM. This constructs a bi-directional feature interaction flow that combines top-down and bottom-up information, effectively preserving fine texture details while reinforcing cross-level semantic transmission, thereby improving the detection accuracy of small objects.
3. Focaler-MPDIoU localization optimization loss: To alleviate the issues of sample imbalance and localization instability in aerial images, a Focaler-MPDIoU loss is introduced. It assigns higher weights to hard samples and incorporates corner-geometry constraints, improving both regression accuracy and localization robustness.
4. Dual-version model design: Two versions, EABI-DETRv1 and EABI-DETRv2, share the same overall architecture but differ in the detection-head channel size. EABI-DETRv1 uses fewer channels to balance lightweight design and real-time performance, suitable for edge deployment, while EABI-DETRv2 employs more channels to enhance feature representation and achieve higher accuracy in complex aerial scenes.

Experiments on the VisDrone2019 dataset show that EABI-DETRv2 achieves 53.4% mAP@0.5 and 34.1% mAP@0.5:0.95, surpassing RT-DETR by 6.2% and 5.1%, respectively. Further evaluations on the HIT-UAV and NWPU VHR-10 datasets confirm its strong generalization, demonstrating robustness and transferability across diverse aerial scenarios. Overall, EABI-DETR achieves an effective balance between lightweight design, accuracy, and scalability, offering a promising solution for UAV-based small object detection.

## 2. Related Work

### 2.1. UAV Object Detection

Small object detection in UAV imagery remains challenging due to tiny target sizes, dense distributions, and strong background interference. Traditional detectors often struggle in long-range, high-density, or occluded scenes, where small targets are easily confused with background textures, resulting in performance degradation. To overcome these issues, recent studies have focused on optimizing detection frameworks. Wang et al. [10] proposed UAV-YOLOv8 with a BiForm attention mechanism to enhance multi-scale representation. Xiao et al. [11] designed SOD-YOLO with a dedicated small-object detection head for complex scenes, while Zhang et al. [12] improved feature fusion through the PARE-YOLO neck redesign. Kong et al. [18] introduced Drone-DETR, integrating a lightweight ESDNet with an EDF-FAM attention module to jointly model global and local features. Zhang et al. [19] further proposed UAV-DETR, leveraging multi-scale fusion and frequency enhancement for improved aerial detection. Although these methods enhance detection accuracy via attention and multi-scale fusion, they still rely heavily on local convolutional modeling, limiting their ability to capture high-resolution details and global context. Moreover, most approaches prioritize accuracy over efficiency and real-time performance. Thus, achieving effective global modeling and multi-scale feature fusion under an efficient and lightweight architecture remains a critical challenge in UAV small object detection.

### 2.2. Attention Mechanisms

Due to their flexible structural design, attention mechanisms can be easily integrated into CNN backbones to enhance discriminative feature learning, thereby attracting significant attention in the computer vision community [20]. The core idea of attention is to adaptively assign weights to emphasize informative features and suppress redundant ones, thus improving the model’s representational capability. Based on the operational dimension, attention mechanisms can be broadly categorized into channel attention, spatial attention, and joint attention. As an early representative, the SE [21] recalibrates channel responses through global average pooling, substantially enhancing semantic aggregation but neglecting spatial structure information. CBAM [22] extends this idea by introducing a spatial branch for cross-dimensional dependency modeling, though its pooling operations add computational overhead. To reduce complexity, SGE [23] introduces a group-wise feature enhancement strategy to improve parameter efficiency and semantic expressiveness, but it still fails to fully model the interaction between spatial and channel dimensions. To address this, SA [24] divides channels into multiple groups and models channel–spatial correlations in two parallel branches through a balanced allocation strategy, improving feature expressiveness. However, its partial channel interaction still limits complete information fusion. CA [25] embeds spatial information into channel attention via a direction-aware mechanism and applies channel reduction to lower computational cost. Nevertheless, the dimensionality reduction causes semantic loss, especially affecting fine-grained feature representation. In contrast, EMA [20] employs a parallel topology with grouped convolutions to jointly capture channel and spatial dependencies, enabling multi-scale contextual aggregation under low computational overhead. It achieves a superior trade-off between performance and efficiency, making it particularly suitable for lightweight detection models embedded in UAV and aerial vision systems.

### 2.3. Multi-Scale Feature Fusion

Multi-scale feature fusion is crucial for detecting objects of varying sizes. Early methods primarily relied on high-level features for prediction, which often led to the loss of fine-grained details from shallow layers. To address this limitation, Liu et al. [26] proposed the Feature Pyramid Network (FPN), which introduces a top-down pathway to propagate semantic information and enable multi-level feature fusion. However, its unidirectional structure restricts information flow, resulting in insufficient utilization of spatial details from lower layers. Subsequently, Liu et al. [27] developed PANet, which adds a bottom-up path aggregation module on top of FPN, allowing the integration of high-level semantics with low-level spatial features. NAS-FPN [28] further improves detection performance through neural architecture search, yet its irregular topology hinders interpretability and generalization. Building upon PANet, BiFPN [29] introduces a bidirectional weighted fusion mechanism, which achieves dynamic and efficient feature aggregation across scales with low computational cost, thereby significantly enhancing object detection accuracy.

### 2.4. Loss Function

In object detection, IoU (Intersection over Union)-based loss functions are widely employed for bounding box regression, aiming to measure the overlap between predicted and ground-truth boxes. Traditional IoU and GIoU [30] often suffer from gradient vanishing when dealing with small objects or high-IoU samples, which limits regression precision. To overcome these limitations, DIoU [31] and CIoU [32] incorporate center-point distance and aspect ratio constraints, improving localization stability, particularly for distant or irregularly shaped objects. However, these methods pay insufficient attention to hard samples under class imbalance conditions. The introduction of Focal Loss alleviates this issue by applying a dynamic weighting strategy that emphasizes difficult samples during optimization. Further, Focaler-IoU [33] combines Focal Loss with the IoU mechanism, adaptively weighting samples of different difficulty levels to enhance overall detection performance. Meanwhile, MPDIoU (Multi-Path Distance IoU) [34] introduces multi-path corner-distance constraints to more precisely characterize the geometric relationship between predicted and ground-truth boxes, demonstrating significant advantages for small-object localization. By integrating the strengths of Focaler-IoU and MPDIoU, the proposed Focaler-MPDIoU [35] jointly addresses hard-sample mining and geometric boundary constraints, effectively improving regression accuracy and robustness.

## 3. Method

**Figure 2:**
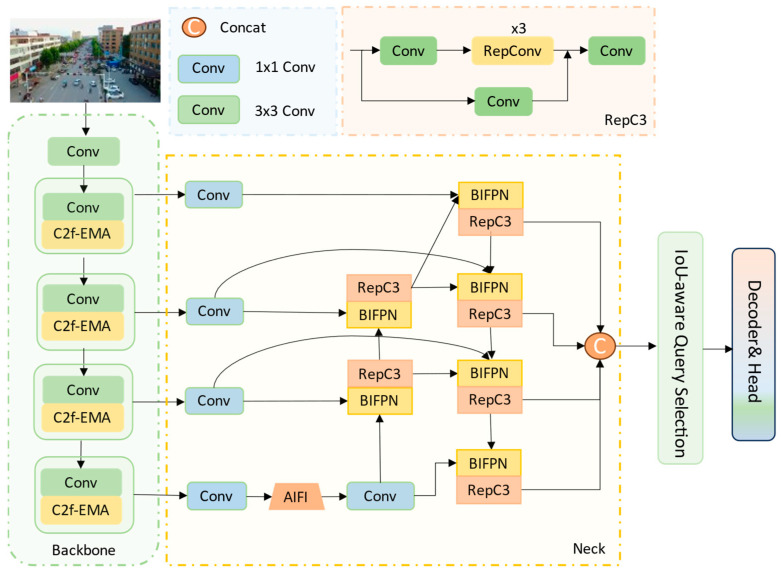
EABI-DETR Network Architecture Diagram.

As illustrated in Figure 2, the proposed EABI-DETR network comprises three main components: Backbone, Neck, and Decoder & Head. Compared with RT-DETR (Figure 1), it introduces key improvements to enhance multi-scale representation and small-object perception. The C2f-EMA module replaces the original BasicBlock in the backbone, employing an efficient multi-scale attention mechanism to strengthen shallow feature extraction. The P2-BiFPN module in the neck substitutes the original CCFM, achieving bidirectional fusion of shallow high-resolution and deep semantic features. Moreover, the Focaler-MPDIoU loss replaces GIoU to better optimize hard samples and improve localization stability. Two variants are developed: EABI-DETRv1 is designed for lightweight and real-time detection on resource-constrained platforms such as UAVs, while EABI-DETRv2 achieves higher detection accuracy while maintaining efficient inference, making it suitable for high-precision aerial applications such as disaster response and traffic monitoring.

### 3.1. C2f-EMA

In the RT-DETR network, the backbone typically employs a feature extraction module based on BasicBlock. Derived from ResNet-18, BasicBlock utilizes two 3 × 3 convolutional layers to perform residual feature learning, offering good computational efficiency and training stability. However, due to the limited receptive field of convolutions, its single-scale feature extraction and the absence of an explicit attention mechanism make it difficult to effectively distinguish foreground objects from complex backgrounds, thereby constraining detection performance.

To enhance small-object perception and model efficiency, this study adopts the lightweight design philosophy of the YOLO family and introduces the C2f module in the feature extraction stage. Nevertheless, features extracted solely through convolution operations remain single-scale and lack sufficient saliency. To address this limitation, an Efficient Multi-scale Attention (EMA) mechanism is embedded within the C2f module, forming a new backbone structure termed C2f-EMA. This design achieves multi-scale feature enhancement and cross-spatial dependency modeling without increasing computational cost.

**Figure 3:**
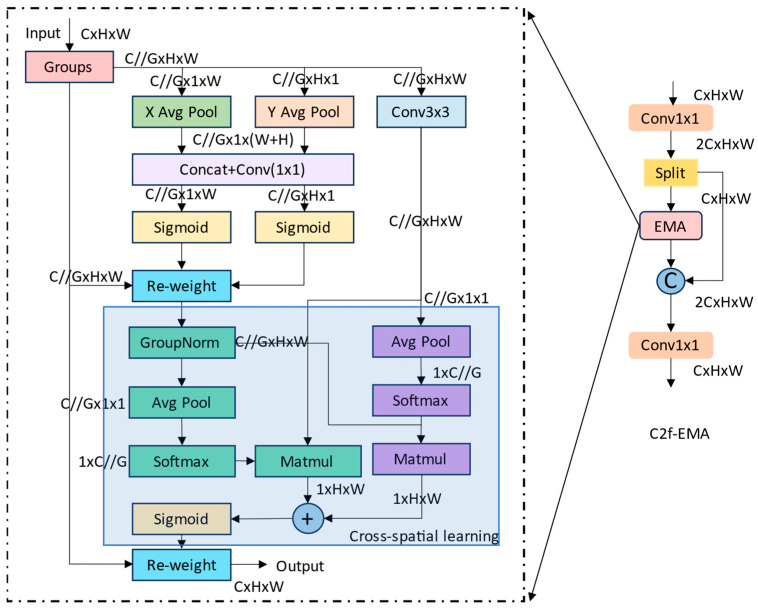
Structure of the C2f-EMA The EMA module consists of grouped processing (Groups), parallel attention branches, and a cross-spatial learning module. It is designed for multi-scale feature fusion and pixel-level recalibration. Here, C denotes the number of channels, H and W represent the height and width of the feature map, and G indicates the number of channel groups.

As illustrated in Figure 3, the C2f-EMA adopts a dual-branch topology: one branch outputs features via a shortcut connection, while the other passes through the EMA module to extract deep semantic representations. The two outputs are then concatenated to achieve complementary fusion of semantic and detailed information. Unlike RT-DETR, which employs two BasicBlocks per stage for feature extraction, such a configuration imposes high computational complexity in shallow layers. To achieve a balance between representation capability and computational efficiency, the proposed model utilizes one C2f-EMA module in stages S2–S4 and three stacked modules in stage S5. Furthermore, only one EMA block is embedded within each C2f bottleneck, enhancing feature representation while avoiding redundant computation.

The C2f-EMA module is designed to enhance the perception and representation of small objects under a lightweight architecture. To balance computational efficiency and feature modeling capacity, the EMA mechanism first divides the input feature map $X\in {\mathbb{R}}^{B\times C\times H\times W}$ into G groups along the channel dimension. Each group is processed independently within its subspace, thereby improving computational efficiency and reducing memory overhead.

In the parallel attention branch, EMA performs multi-scale feature modeling through two 1 × 1 branches and one 3 × 3 branch. The two 1 × 1 branches conduct global average pooling (GAP) along the height and width directions, respectively, to capture the channel-wise statistical characteristics in both spatial dimensions. The resulting features are concatenated and passed through a 1 × 1 convolution followed by dual Sigmoid activations, generating a direction-aware channel attention map that emphasizes salient regions while suppressing irrelevant channels. Meanwhile, the 3 × 3 branch models spatial contextual dependencies, producing a fine-grained spatial attention map that complements the channel attention mechanism.

The Sigmoid activation function is defined as:

(1) $$\sigma (x)=\frac{1}{1+e^{-x}}$$

This function maps the input feature value x to the range [0, 1], highlighting salient features while suppressing irrelevant ones, where e denotes the natural constant.

During the cross-spatial learning stage, EMA first applies Group Normalization (GroupNorm) to mitigate inter-group distributional discrepancies and stabilize training. Then, average pooling, softmax, and matrix multiplication (MatMul) operations are employed to construct spatial dependency relationships. Afterward, Sigmoid activation and feature reweighting (Re-weight) operations are applied for pixel-level feature enhancement, enabling the model to focus on discriminative regions while suppressing background noise.

The Softmax function is formulated as:

(2) $$\mathrm{Softmax}(x_{i})=\frac{e^{x_{i}}}{\sum_{j=1}^{n}e^{x_{j}}}$$

This function normalizes the input vector into a probability distribution, representing the relative importance of each element $x_{i}$, where n denotes the dimension of the input vector.

Overall, the C2f-EMA module significantly strengthens feature representation and spatial perception through multi-branch fusion and multi-scale attention mechanisms. Compared with the BasicBlock, it captures fine-grained details and key semantic regions more effectively, achieving superior accuracy and computational efficiency in small object detection particularly suitable for resource-constrained aerial applications.

### 3.2. P2-BiFPN

In object detection tasks, crucial spatial details are primarily encoded in the shallow layers of a convolutional network. However, these details are progressively weakened or even lost through successive convolutions and downsampling operations, leading to insufficient feature representation for small objects. Moreover, during feature extraction, small objects can be easily occluded by larger ones or interfered with by background noise, resulting in further degradation of fine-grained information.

**Figure 4:**
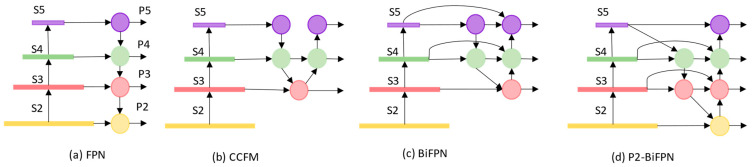
Overview of pyramid feature fusion networks. (**a**) FPN uses a top-down fusion path; (**b**) CCFM adopts an inverted-pyramid strategy; (**c**) BiFPN performs bidirectional fusion with learnable weights; (**d**) P2-BiFPN adds shallow high-resolution features (P2) to enhance small-object detection. S2–S5 represent feature maps from stages 2–5 of the backbone.

As illustrated in Figure 4, the traditional Feature Pyramid Network (FPN) integrates multi-scale information through a top-down feature fusion pathway and performs detection based on the fused high-level semantic features. Although this structure improves multi-scale representation, its unidirectional information flow causes shallow details to weaken after multiple downsampling operations, limiting the preservation of fine spatial textures crucial for small object detection and thus constraining detection performance.

To address this issue, RT-DETR introduces the CCFM, which adds a bottom-up aggregation path to FPN, thereby achieving bidirectional interaction between high- and low-level features. CCFM adopts an “inverted pyramid” fusion strategy, in which high-resolution features are downsampled and low-resolution features are upsampled to achieve alignment and fusion at intermediate scales. This design effectively enhances the joint modeling of semantics and detailed representations. However, its detection heads are only applied to the P3–P5 scales, neglecting the shallow high-resolution features (e.g., from the S2 layer). Consequently, the fusion process still focuses mainly on mid- and high-level features, resulting in limited fine-grained feature representation a deficiency that becomes particularly pronounced in aerial scenes with densely distributed small objects.

Bidirectional Feature Pyramid Network (BiFPN) further refines the multi-scale fusion process by introducing bidirectional pathways (top-down and bottom-up) and learnable weighting mechanisms, allowing the network to adaptively assign importance across different scales. This facilitates a more balanced integration of semantic and detailed information. In addition, BiFPN directly connects higher-level semantic features (from S4 and S5) with shallower layers, enhancing inter-level correlation and mitigating information loss during deep propagation. Nevertheless, BiFPN still underutilizes high-resolution features from the P2 layer. Directly incorporating P2 features can introduce redundancy and computational overhead, which compromises real-time performance and lightweight design key requirements for UAV-based or resource-constrained platforms.

To overcome these limitations, this study uses the P2-BiFPN module. This module explicitly integrates shallow, high-resolution P2 features into the BiFPN framework and employs a dedicated small-object detection head to improve the perception of small targets. Structurally, P2-BiFPN streamlines the high-level branches by removing redundant P5 layers and performing efficient fusion between shallow and deep features at intermediate levels. This design significantly reduces computational complexity while maintaining effective multi-scale feature interaction. Through multi-level bidirectional feature propagation and adaptive weighted fusion, P2-BiFPN promotes stronger collaboration between shallow spatial details and deep semantic representations, compensating for the deficiencies of RT-DETR in small object detection. This design not only enhances multi-scale feature modeling capability but also achieves an excellent balance between accuracy and efficiency, making it particularly suitable for real-time deployment on UAV and other mobile platforms.

### 3.3. Loss Function

#### 3.3.1. Focaler-IoU

In traditional IoU-based regression, all samples are optimized equally, disregarding the importance of hard samples during the regression process. Focaler-IoU addresses this issue by introducing a weighted reconstruction over the IoU range, enabling the model to focus more on small and difficult-to-regress targets during training, thereby improving the accuracy of boundary fitting. Specifically, a piecewise weighting function is defined over the IoU values as follows:

(3) $$IoU_{\mathrm{focaler}}=\left\{\begin{array}{ll} 0, & IoU<d,\\ \frac{IoU-d}{u-d}, & d\leq IoU\leq u,\\ 1, & IoU>u \end{array}\right.$$

Here, $IoU^{\mathrm{focaler}\;}$ is a reconstruction of IoU. $\left[d,u\right]\in [0,1]$ denotes a tunable weighting range that controls the relative importance of samples across different IoU stages. When the IoU value is low (i.e., the predicted box has limited overlap with the ground truth), the model assigns a higher weight to emphasize hard samples and enhance their optimization. Conversely, for easily detectable samples, the weight is appropriately reduced to mitigate overfitting. The corresponding loss function is defined as follows:

(4) $$L_{\mathrm{Focaler}-\mathrm{IoU}}=1-IoU_{\mathrm{focaler}}$$

This design employs a piecewise linear scheduling mechanism to achieve adaptive weighting between hard and easy samples, effectively alleviating the training bias caused by sample imbalance in small object detection.

#### 3.3.2. MPDIoU

**Figure 5:**
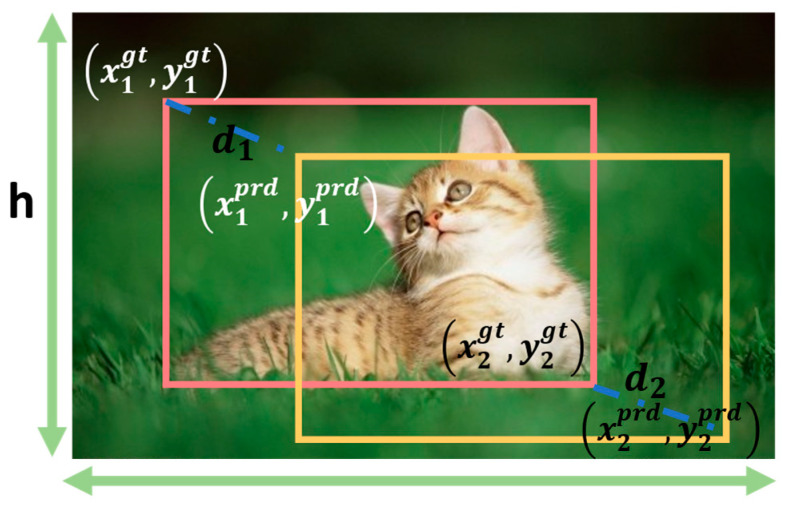
Geometric Illustration of MPDIoU.

Although improved IoU-based losses such as DIoU and CIoU introduce constraints on boundary overlap and the distance between center points, they fail to optimize cases where the predicted and ground-truth boxes share the same aspect ratio but differ significantly in absolute width and height. To address this limitation, Ma et al. [34] proposed the Multi-Path Distance IoU (MPDIoU), a similarity metric that reinforces spatial consistency in bounding box regression by introducing geometric distance constraints. As illustrated in Figure 5, let $d_{1}$ and $d_{2}$ denote the distances between the top-left and bottom-right corners of the predicted and ground-truth boxes, respectively. Then, MPDIoU is defined as:

(5) $$\mathrm{MPDIoU}=\frac{B_{\mathrm{gt}}\cap B_{\mathrm{prd}}}{B_{\mathrm{gt}}\cup B_{\mathrm{prd}}}-\frac{d_{1}^{2}}{w^{2}+h^{2}}-\frac{d_{2}^{2}}{w^{2}+h^{2}},$$

(6) $$L_{\mathrm{MPDIoU}}=1-\mathrm{MPDIoU}$$

Here, w and h represent the width and height of the image, respectively, while $B_{\mathrm{gt}\;}$ and $B_{\mathrm{prd}\;}$ denote the ground-truth box (red box) and the predicted box (yellow box). This design introduces corner-point distance constraints, which simultaneously account for shape proportions and spatial positional differences during the computation of overlapping regions. As a result, it achieves greater stability and geometric consistency in the regression of small and hard-to-detect objects.

#### 3.3.3. Focaler-MPDIoU

The Focaler-MPDIoU loss function used in this study simultaneously considers sample difficulty weighting and geometric structure constraints. Built upon the Focaler-IoU framework, it introduces the MPDIoU distance constraint, which focuses on hard samples through an adaptive weighting mechanism while leveraging corner-point geometric relationships to enhance localization robustness. The loss function is defined as follows:

(7) $$L_{\mathrm{Focaler}-\mathrm{MPDIoU}}=L_{\mathrm{MPDIoU}}+\mathrm{IoU}-{\mathrm{IoU}}^{\mathrm{Focaler}}$$

## 4. Experiment and Results

### 4.1. Datasets

In this paper, we conduct experiments on three datasets: VisDrone2019, HIT-UAV, and NWPU VHR-10.

**Figure 6:**
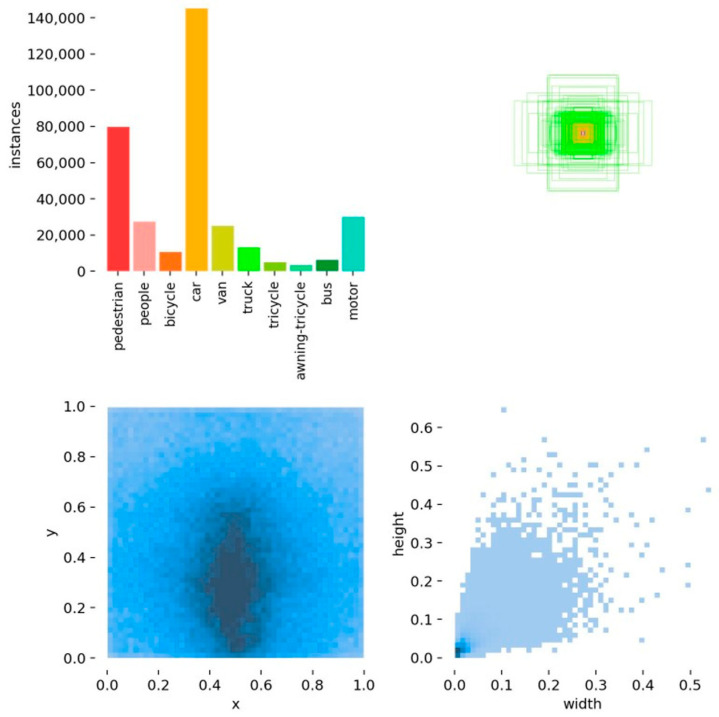
Visualization of the VisDrone2019 dataset.

The VisDrone2019 dataset contains 8629 images captured from various locations and altitudes, covering 10 object categories. Among them, 6471 images are used for training, 548 for validation, and 1610 for testing. The dataset features a large number of small objects with highly imbalanced size distributions, closely reflecting real-world aerial scenarios. As shown in Figure 6, the annotation statistics and distribution characteristics of VisDrone2019 are as follows: the top-left subfigure presents the number of instances per class, showing a distinct imbalance—car instances dominate, followed by pedestrian and motor; the top-right subfigure illustrates the prior distribution of bounding box scales, revealing the concentration regions of object sizes across different categories. The bottom-left subfigure displays the heatmap of object center coordinates (x,y), where most objects are clustered in the lower central region of the images; the bottom-right subfigure depicts the normalized (width,height) distribution, indicating that most objects have widths and heights smaller than 0.2, confirming that the dataset is dominated by small targets with significant scale variation.

The HIT-UAV dataset consists of 2898 infrared thermal images extracted from 43,470 video frames, covering diverse scenes such as campuses, parking lots, and roadways. It includes objects of various categories, flight altitudes, camera angles, and illumination conditions, making it particularly suitable for evaluating detection performance under dynamic and illumination-varying environments.

The NWPU VHR-10 dataset is a high-resolution remote sensing benchmark designed for object detection. It contains 800 images with 10 object categories (e.g., airplanes, vehicles, harbors), comprising 3651 annotated instances in total. This dataset is widely used to assess the detection capability of models in complex backgrounds and for small objects in remote sensing imagery.

### 4.2. Experiment Environment and Training Strategy

In this study, RT-DETR-R18 is adopted as the baseline model for improvement and performance evaluation. Compared with other RT-DETR variants, this model features a more lightweight architecture and lower computational complexity, making it particularly suitable for deployment on resource-constrained embedded platforms such as UAVs. All experiments are conducted on a Linux system using an NVIDIA RTX 4090D GPU (24 GB), implemented with PyTorch 2.1.1 and CUDA 12.1. The training is performed for 200 epochs with a batch size of 4, using the AdamW optimizer with a learning rate of 0.0001 and momentum of 0.9. The input image resolution is set to 640 × 640 pixels.

### 4.3. Evaluation Metrics

To evaluate the detection performance of our enhanced model, the following evaluation metrics were used in this study:

Precision: Measures the proportion of true positives among all predicted positive samples. Its definition is given by Formula (8):

(8) $$\mathrm{Precision}=\frac{TP}{TP+FP}$$

where True Positives (*TP*) are the number of instances correctly predicted as positive, False Positives (*FP*) are the number of instances incorrectly predicted as positive, and False Negatives (*FN*) are the number of instances missed by the model as positive.

Recall: Measures the proportion of actual positive samples correctly predicted. Its definition is given by Formula (9):

(9) $$\mathrm{Recall}=\frac{TP}{TP+FN}$$

Average Precision (*AP*): Refers to the area under the precision-recall curve and is used to evaluate the performance of single-class detection. Its definition is given by Equation (10):

(10) $$AP=\int_{0}^{1}\mathrm{Precision}(\mathrm{Recall})d(\mathrm{Recall})$$

MAP (mean Average Precision) represents the average *AP* value across all classes and is used to measure the overall detection performance of the model across the entire dataset. Its definition is given by Formula (11):

(11) $$mAP=\frac{1}{N}\sum_{i=1}^{N}AP_{i}$$

where ${AP}_{i}$ represents the average precision of the i-th class, and N represents the total number of classes in the training set. $mAP_{0.5}$ represents the average precision at an IoU threshold of 0.5, and $mAP_{0.5:0.95}$ represents the average precision across IoU thresholds ranging from 0.5 to 0.95 (with a step size of 0.05).

In addition, model complexity, computational overhead, and inference speed were evaluated using the number of parameters (Params), GFLOPs, and frames per second (FPS).

### 4.4. Experiment Results

#### 4.4.1. Comparison Experiments on VisDrone2019

The comparative experimental results on the VisDrone2019 dataset (as shown in Table 1) demonstrate that the proposed EABI-DETR model achieves outstanding performance in UAV-based small object detection tasks. Compared with the baseline RT-DETR-R18, EABI-DETRv2 improves mAP@0.5 and mAP@0.5:0.95 by 6.2% and 5.1%, respectively, indicating superior detection capability. Meanwhile, it maintains a computational complexity of 85.7 GFLOPs and 13.69 M parameters, significantly reducing model size while preserving high detection accuracy—highlighting its efficiency and lightweight characteristics. In terms of inference speed, EABI-DETRv2 achieves 97.5 FPS, which is slightly lower than the baseline model but demonstrates a better trade-off between accuracy, speed, and computational cost compared with high-precision detectors such as UAV-DETR-R50, AMFEF-DETR, and RT-DETR-R50. Furthermore, EABI-DETRv2 consistently outperforms all YOLO-series models, achieving higher detection accuracy.

Notably, the lightweight version EABI-DETRv1, with only 9.88 M parameters and 51.9 GFLOPs, attains an impressive 109.1 FPS, maintaining real-time performance while preserving competitive detection accuracy. This demonstrates its exceptional efficiency and strong potential for practical deployment in UAV-based visual applications.

Table 2 presents the detection performance of the EABI-DETRv2 model across different object categories on the VisDrone2019 dataset. Overall, the model achieves a Precision of 65.2% and a Recall of 51.3% on the validation set, with mAP@0.5 and mAP@0.5:0.95 reaching 53.4% and 34.1%, respectively. These results demonstrate the model’s strong capability in multi-class and multi-scale object detection from aerial imagery, particularly under complex background conditions where it exhibits remarkable adaptability. Specifically, EABI-DETRv2 achieves outstanding detection accuracy in the car, bus, and pedestrian categories, with Precision values of 83.0%, 83.2%, and 71.6%, and corresponding mAP@0.5 scores of 88.0%, 72.3%, and 61.7%, respectively. Furthermore, for categories with higher intra-class variability or frequent occlusion such as people, van, and motor the model maintains robust performance, achieving mAP@0.5 scores of 53.4%, 55.4%, and 65.0%, respectively. Although the detection performance on certain small-object categories, such as bicycle and awning-tricycle, is relatively lower (with mAP@0.5 of 28.8% and 21.6%, respectively), these results still highlight the model’s potential in handling complex-shaped and low-texture objects.

In summary, EABI-DETR balances accuracy and robustness in multi-class detection tasks in aerial images, demonstrating excellent adaptability and practical value.

**Figure 7:**
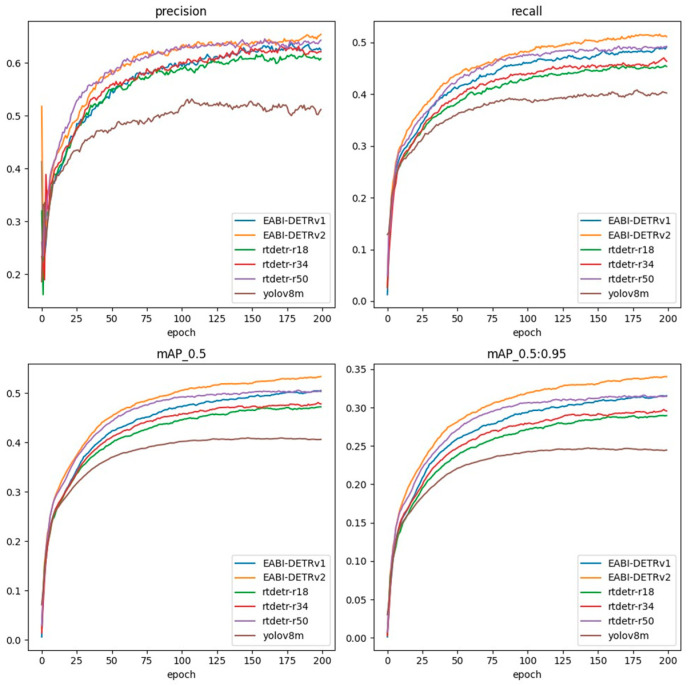
Training convergence comparison on the VisDrone2019 dataset.

Figure 7 illustrates the variations in Precision, Recall, mAP@0.5, and mAP@0.5:0.95 during the training process for different models. The results indicate that EABI-DETRv2 consistently outperforms other models across all metrics, exhibiting faster convergence and higher final accuracy. Meanwhile, EABI-DETRv1 also surpasses the baseline model, maintaining strong detection performance with substantially lower computational complexity.

**Figure 8:**
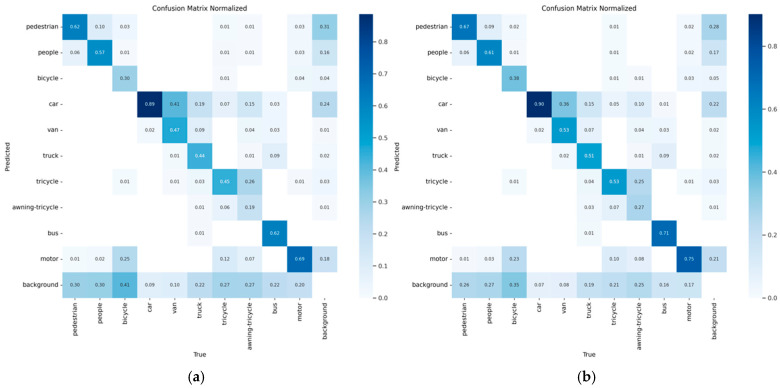
(**a**) Confusion Matrix of RT-DETR; (**b**) Confusion Matrix of EABI-DETRv2.

Figure 8 shows a comparison of the confusion matrices between EABI-DETRv2 and RT-DETR under the same parameter settings. The results indicate that EABI-DETRv2 achieves a higher correct classification rate across all object categories, while effectively reducing false positives and false negatives. Particularly in dense small object categories, such as bicycle and tricycle, the true positive rate increases by approximately 8% compared to RT-DETR. The model also shows a similar improvement in difficult categories with occlusion features, such as awning-tricycle. These results fully demonstrate the advantages of EABI-DETRv2 in small object detection and complex scene perception, validating its potential application in real-world drone image detection tasks.

As shown in Table 3, several IoU-based loss functions were compared under identical experimental settings. The results indicate that although EIoU and MPDIoU outperform GIoU in terms of localization accuracy, they still exhibit suboptimal boundary fitting for small objects. In contrast, Focaler-MPDIoU, which integrates the sample-weighting strategy of the Focal mechanism with the corner-point distance constraint of MPDIoU, achieves the best performance with mAP@0.5 = 50.5% and mAP@0.5:0.95 = 31.5%. These results demonstrate that Focaler-MPDIoU effectively enhances both the detection accuracy and regression stability for small objects, and is therefore adopted as the final regression loss function in this study.

#### 4.4.2. Ablation Experiment

To evaluate the contribution of each component to the overall model performance, an ablation experiment was conducted on EABI-DETRv2 by progressively integrating individual enhancement modules into the baseline RT-DETR-R18 model and observing the resulting performance variations. The experimental results are summarized in Table 4. First, the individual introduction of the C2f-EMA and P2-BiFPN modules each leads to noticeable improvements in detection performance, with gains of 1.0% and 0.9%, respectively, while also reducing the parameter count by 37.6% and 12.1%. When both modules are combined, the model’s Precision and Recall increase to 64.7% and 51.8%, respectively, and the mAP@0.5 and mAP@0.5:0.95 reach 52.9% and 33.5%. Finally, after integrating all modules, the complete EABI-DETR model achieves Precision, Recall, mAP@0.5, and mAP@0.5:0.95 values of 65.2%, 51.3%, 53.4%, and 34.1%, respectively, representing improvements of 4.4%, 5.9%, 6.2%, and 5.1% over the baseline model. Moreover, the parameter count is reduced to 13.69 M, demonstrating that the proposed modules not only enhance detection accuracy but also maintain computational efficiency. Although the introduction of multi-scale fusion structures and attention mechanisms slightly reduces the inference speed (FPS) compared with the baseline, the overall performance remains high, confirming that EABI-DETRv2 achieves a well-balanced trade-off between detection accuracy and real-time performance.

As shown in Table 5, to analyze the impact of parameter configurations on model performance, we adjusted the corner penalty normalization factor $w^{2}+h^{2}$ and the Focaler interval parameters (d,u). The results indicate that when $\;w^{2}+h^{2}$ = 2, the overall model performance surpasses that with $w^{2}+h^{2}$ = 1, suggesting that moderately increasing the normalization coefficient effectively reduces excessive penalties on small targets and improves regression stability. Meanwhile, under the same normalization coefficient, the configuration $\left(d,u\right)=(0.00,0.95)$ yields the best performance with mAP@0.5 = 50.5%. Although $\left(d,u\right)=(0.05,0.97)$ slightly improves mAP@0.5:0.95, this interval causes the model to overly focus on high-IoU samples, weakening its learning effectiveness on small targets with medium or low IoU values. Considering both detection accuracy and small-object performance, the optimal configuration of the Focaler-MPDIoU loss function is determined as $\left(w^{2}+h^{2},d,u\right)=(2,0.00,0.95)$.

#### 4.4.3. Generalization Experiment

To validate the generalization capability of the proposed EABI-DETR model in small object detection scenarios, experiments were conducted on the HIT-UAV and NWPU VHR-10 datasets.

On the HIT-UAV dataset (see Table 6), EABI-DETRv2 achieved mAP@0.5 = 81.9% and mAP@0.5:0.95 = 53.3%, outperforming other models in the DETR series, including RT-DETR-R18, RT-DETR-R34, and RT-DETR-R50. Although YOLOv8m attains slightly higher accuracy, it contains 25.85 M parameters nearly twice that of EABI-DETRv2 (13.69 M). Overall, EABI-DETRv2 demonstrates a superior balance between detection accuracy, parameter efficiency, and computational complexity, indicating its strong detection precision and generalization capability.

The experimental results on the NWPU VHR-10 dataset (as shown in Table 7) demonstrate that EABI-DETRv2 achieves detection performance of mAP@0.5 = 89.5% and mAP@0.5:0.95 = 68.0%, outperforming comparison models such as YOLOv8m and RT-DETR-R18. Although some more complex models (e.g., RT-DETR-R34) show slight improvements in individual metrics, EABI-DETRv2 attains comparable or even superior detection accuracy with significantly lower computational complexity (85.7 GFLOPs) and a smaller number of parameters (13.69 M). These results highlight the model’s strong adaptability and computational efficiency in high-resolution remote sensing imagery, further validating the robustness and generalization capability of the proposed approach across different scenarios.

#### 4.4.4. Visualization

**Figure 9:**
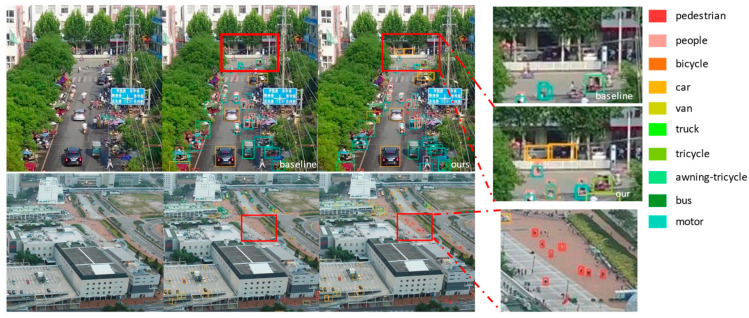
Visualization Analysis Results of RT-DETR-R18 and EABI-DETRv2.

Figure 9 shows the detection visualization comparison between RT-DETR-R18 and the proposed EABI-DETRv2 on the VisDrone2019 dataset. The regions highlighted by the red boxes indicate that EABI-DETR demonstrates stronger detection capability in dense and heavily occluded aerial scenes, capturing more and farther small objects while significantly improving localization accuracy. Compared to the baseline model, EABI-DETR performs better in fine-grained recognition, with a noticeable reduction in missed detection rates, and shows good robustness and stability across various complex scenes.

## 5. Conclusions

The proposed EABI-DETR is an efficient end-to-end detection framework designed for small object detection in aerial imagery. Building upon RT-DETR, the model introduces the C2f-EMA module to enhance the joint modeling of channel semantics and spatial structure, and integrates the P2-BiFPN module to achieve efficient fusion of shallow details and multi-scale features. These improvements effectively mitigate the challenges of dense distribution, occlusion, and complex backgrounds commonly encountered in aerial scenes. Moreover, the Focaler-MPDIoU loss function further improves the boundary regression accuracy and robustness for hard samples. Experimental results demonstrate that EABI-DETR achieves significant performance gains on public benchmarks, including VisDrone2019, HIT-UAV, and NWPU VHR-10, surpassing state-of-the-art methods in detection accuracy, localization stability, and adaptability to complex environments.

The model also exhibits strong application potential in pedestrian monitoring, vehicle recognition, and low-altitude security surveillance, making it particularly suitable for aerial monitoring and disaster response scenarios where accuracy is prioritized over real-time performance.

However, EABI-DETR still faces certain limitations in practical applications. Due to the incorporation of multi-scale fusion and attention mechanisms, its inference speed (FPS) is slightly lower than that of some lightweight detectors, leaving room for improvement in latency-sensitive scenarios such as real-time UAV surveillance and traffic analysis. In addition, detection accuracy may still degrade when objects are extremely small or when backgrounds are highly cluttered. Future research will focus on further enhancing model efficiency and adaptive fusion mechanisms through network pruning, knowledge distillation, and dynamic attention strategies to improve real-time performance. Moreover, by integrating multi-task learning and cross-modal feature fusion, future work aims to extend the applicability of EABI-DETR to intelligent transportation, emergency monitoring, and urban perception, advancing UAV-based small object detection toward higher accuracy, efficiency, and reliability.

## Figures and Tables

**Table 1:** Comparison Experiment on the VisDrone2019-Val Dataset.

Models	mAP_0.5_/%	mAP_0.5:0.95_/%	GFLOPs	Params/M	FPS
SOD-UAV [36]	45.7	26.8	126.2	32.3	-
SOD-YOLO [11]	50.7	30	83.5	30.3	72.5
PARE-YOLO [12]	46.3	28.4	45.1	26.3	123.5
Drone-DETR [18]	53.9	33.9	128.3	28.7	30
UAV-DETR-R50 [19]	51.1	31.5	170	42	65
AMFEF-DETR [37]	51.87	32.27	142	35.81	84.5
RT-DETR-R18 [16]	47.2	29.0	57.0	19.88	135.1
RT-DETR-R34	48.1	29.7	88.8	31.12	102.8
RT-DETR-R50	50.6	31.7	129.6	41.97	74.7
EABI-DETRv1	50.5	31.5	51.9	9.88	109.1
EABI-DETRv2 (ours)	53.4	34.1	85.7	13.69	97.5

**Table 2:** Experimental Results of the EABI-DETRv2 Model on Various Categories of the VisDrone2019 Dataset.

Class	Precision/%	Recall/%	mAP_0.5_/%	mAP_0.5:0.95_/%
All	65.2	51.3	53.4	34.1
pedestrian	71.6	56.5	61.7	32.5
people	67.8	50.3	53.4	23.8
bicycle	50.9	29.4	28.8	14.4
car	83.0	85.0	88.0	65.8
van	70.4	51.3	55.4	42.6
truck	66.1	44.8	46.1	32.1
tricycle	52.9	43.8	41.2	25.2
awning-tricycle	39.4	21.4	21.6	14.6
bus	83.2	67.7	72.3	55.8
motor	66.6	62.9	65.0	33.6

**Table 3:** Comparative experiments of EABI-DETRv1 with different loss functions.

IOU	mAP_0.5_/%	mAP_0.5:0.95_/%
GIOU	49.1	30.5
EIOU [38]	50.2	31.4
Focaler IoU	49.2	30.3
MPDIoU	49.7	30.9
Focaler-MPDIoU	50.5	31.5

**Table 4:** Ablation Experiment of EABI-DETRv2.

C2f-EMA	P2-BIFPN	Focaler-MPDIoU	Precision	Recall	mAP_0.5_	mAP_0.5:0.95_	Params/M	FPS
✓	×	×	62.5	46.1	48.2	29.7	12.4	119.3
×	✓	×	61.7	46.8	48.1	29.8	17.47	125.7
✓	✓	×	64.7	51.8	52.9	33.5	13.69	97.2
✓	✓	✓	65.2	51.3	53.4	34.1	13.69	97.5

Note: ✓ indicates that the module is used, and × indicates that the module is not used.

**Table 5:** Comparative experiments of Focaler-MPDIoU with different parameter settings.

$w^{2}+h^{2}$	*d*	*u*	mAP_0.5_/%	mAP_0.5:0.95_/%
1	0.00	0.95	49.9	31.1
1	0.05	0.97	50.0	31.0
2	0.00	0.95	50.5	31.5
2	0.05	0.97	50.3	31.7

**Table 6:** Generalization Experiment of EABI-DETRv2 on the HIT-UAV dataset.

Models	mAP_0.5_/%	mAP_0.5:0.95_/%	GFLOPs	Params/M
YOLOv8m	84.6	56.5	78.7	25.85
RT-DETR-R18	79.8	51.7	57.0	19.88
RT-DETR-R34	78.5	49.6	88.8	31.11
RT-DETR-R50	79.6	51.1	129.6	42.0
EABI-DETRv2 (ours)	81.9	53.3	85.7	13.69

**Table 7:** Generalization Experiment of EABI-DETRv2 on the NWPU VHR-10 Dataset.

Models	mAP_0.5_/%	mAP_0.5:0.95_/%	GFLOPs	Params/M
YOLOv8m	88.9	67.2	78.7	25.85
RT-DETR-R18	87.3	65.8	57.0	19.88
RT-DETR-R34	90.6	68.5	88.8	31.12
RT-DETR-R50	87.3	66.5	129.6	41.97
EABI-DETRv2 (ours)	89.5	68.0	85.7	13.69

## Data Availability

The code and data used in this paper can be obtained from the corresponding author upon reasonable request.
